# Editorial: Exploring immunomodulation to balance maladaptive inflammation and restore tissue homeostasis

**DOI:** 10.3389/fimmu.2025.1723620

**Published:** 2025-10-28

**Authors:** David M. Smadja, Maxime Jeljeli, Alexandre G. Lellouch

**Affiliations:** ^1^ Université Paris Cité, INSERM, PARCC, Paris, France; ^2^ Department of Hematology, AP-HP, Georges Pompidou European Hospital, Paris, France; ^3^ Division of Plastic and Reconstructive Surgery, Cedars Sinai Hospital, Los Angeles, CA, United States

**Keywords:** immunomodulation, COVID-19, sepsis, pulmonary microbiota, hyperthermia

The COVID-19 pandemic reignited global interest in how immune responses can oscillate between hyperinflammation and profound suppression. Severe SARS-CoV-2 infections, marked by thromboinflammatory cascades and immune dysregulation, exemplify the importance of immunomodulation in maintaining tissue homeostasis. The pandemic also opened the way for renewed appreciation of the immune system’s dual role—not only as a defender against pathogens but also as a regulator of repair, resolution, and tolerance.

This Research Topic brings together original research and reviews that collectively explore how immunomodulatory interventions may rebalance inflammation, restore tissue integrity, and prevent immune exhaustion or immune escape. The articles span acute infections such as sepsis and COVID-19, to chronic inflammatory states and tumor immunology, while integrating systemic and local perspectives on immune control.

## Sepsis and immunoparalysis: two sides of immune dysregulation

Two contributions address the complexity of immune dynamics in sepsis. In a prospective study of critically ill patients, Samuelsen et al. examined the trajectories of immunoparalysis biomarkers—including mHLA-DR expression, lymphocyte counts, and cytokine responses—over the first 14 days of illness. They observed diverging recovery patterns between septic and non-septic patients and revealed that ex vivo cytokine stimulation may outperform classical markers in predicting immune recovery. Importantly, while mHLA-DR increased more in non-septic patients, absolute lymphocyte counts and TNF-α production showed more rapid recovery in those with sepsis, suggesting distinct temporal windows for immuno-adjuvant therapy.

Complementing this systemic perspective, Vernay et al. focused on *compartmentalized immunity* in pulmonary sepsis and the lung’s susceptibility to secondary infections. Their comprehensive review emphasizes that systemic immune markers often fail to capture local immune dysfunction in the lung. The authors detail how infection-induced remodeling of alveolar macrophages, altered efferocytosis, microbiota dysbiosis, and regulatory T cell expansion may lead to persistent immunosuppression in the pulmonary microenvironment. These changes, distinct from blood-based markers, call for spatially resolved diagnostics and site-specific interventions.

## Steroids and fungal immunity: when tolerance turns inflammatory

In another infection-related context, Koerber et al. revealed a paradoxical activation of the innate immune system in *dexamethasone-treated tolerogenic dendritic cells* (Dex-DCs) upon fungal stimulation with β-glucans. Although Dex-DCs display hallmarks of immune tolerance, the authors show that Dectin-1 engagement in these cells leads to pronounced NLRP3 inflammasome activation, pyroptosis, and IL-1β/IL-18 release. This inflammasome response was ROS-dependent and could be mitigated by Syk or NLRP3 inhibition. The study highlights an underappreciated risk: corticosteroid therapies, while immunosuppressive, may still permit potent innate activation, posing a threat for patients exposed to fungal pathogens. The work also suggests that NLRP3 inhibitors might play a protective role in such contexts, though risks of dampening host defense must be carefully evaluated.

## Modeling inflammation in COVID-19: systems immunology at work

Extending the lessons of COVID-19 to the systems level, Murad et al. employed a computational modeling framework to investigate how SARS-CoV-2 infection perturbs the hemostatic and complement systems and how different therapeutic strategies modulate these responses. By simulating concentration–time profiles for components such as thrombin, plasmin, and tissue plasminogen activator, the study demonstrates how treatment interventions can either restore or destabilize coagulation–complement balance. The model also suggests that individualized therapies must account for timing and dose-specific effects on thromboinflammation. These insights align with clinical observations during COVID-19, where early or excessive anticoagulation sometimes led to unintended immunologic consequences.

## Immune metabolism and anti-inflammatory programming in macrophages

Chronic inflammation also stems from disrupted metabolic-immune crosstalk. Albany et al. explored how exposure to oxidized LDL (oxLDL), a key driver in atherosclerosis, reprograms macrophage function, surprisingly redirected by regulatory T cells (Tregs). The authors found that Tregs promoted cholesterol efflux and shifted oxLDL-exposed macrophages toward an anti-inflammatory phenotype. Mechanistically, this was associated with increased expression of IL-10, ABCA1, and GATA-3. These findings support a model in which immune-metabolic homeostasis may be restored not by suppressing inflammation broadly, but by enhancing resolution-promoting pathways at the macrophage level.

## Harnessing thermal stress in tumor immunity

Completing the Research Topic, Abreu et al. reviewed the immunological consequences of *hyperthermia therapy* in cancer and its potential to augment checkpoint blockade efficacy. They examine how thermal stress reshapes the tumor immune microenvironment, enhancing antigen presentation, heat shock protein expression, and T cell infiltration. Importantly, hyperthermia may help overcome immune exclusion or exhaustion in poorly immunogenic tumors. The authors advocate for integrating heating with immunotherapies to stimulate innate and adaptive responses synergistically.

## Conclusion: precision immunomodulation for diverse contexts

Across these six contributions, a unifying insight becomes clear: the immune system cannot be simplistically classified as pro- or anti-inflammatory. Rather, its behavior is highly context-dependent, governed by a dynamic interplay of temporal factors, tissue-specific microenvironments, and therapeutic interventions. This complexity becomes especially evident in the diverse immunological landscapes of acute infections, metabolic disorders, malignancies, and systemic inflammatory conditions. Particularly, conditions such as chronic inflammation and vascular dysfunction exemplify how persistent immune dysregulation can lead to pathological remodeling and tissue damage. Similarly, hyperthermia, in an infectious context or induced therapeutically, has demonstrated profound immunomodulatory effects including enhanced antigen presentation, improved immune cell trafficking, and reshaped cytokine responses. These examples highlight the nuanced roles that stressors play in either perpetuating immune imbalance or facilitating its resolution. Collectively, these studies underscore a paradigm shift in immunotherapy, from blunt immune suppression toward precise, adaptive modulation of immune pathways. The goal is not only to dampen inflammation, but to recalibrate the immune response so that it is both effective in eliminating threats and restrained enough to avoid collateral tissue damage. By elucidating the molecular and cellular mechanisms underlying immune imbalance, and showcasing innovative approaches to restoring homeostasis, this Research Topic highlights the growing promise of context-aware, precision immunotherapies. These insights lay the groundwork for developing interventions that are tailored not only to disease type, but also to its specific immunological and physiological context, paving the way for the next generation of personalized medicine.

